# Evaluating the association between socioeconomic position and cardiometabolic risk markers in young adulthood by different life course models

**DOI:** 10.1186/s12889-022-13158-0

**Published:** 2022-04-09

**Authors:** Mia Klinkvort Kempel, Trine Nøhr Winding, Morten Böttcher, Johan Hviid Andersen

**Affiliations:** 1 Department of Occupational Medicine - University Research Clinic, Danish Ramazzini Centre, Goedstrup Hospital, Gl. Landevej 61, 7400 Herning, Denmark; 2 Cardiovascular Research Unit, Department of Cardiology, Gødstrup Hospital, Herning, Denmark

**Keywords:** Socioeconomic position, Social mobility, Lifestyle, Life course models, Cardiometabolic diseases, Epidemiology

## Abstract

**Background:**

Cardiometabolic health in adulthood is associated with socioeconomic position (SEP) in childhood. Although this has been studied by previous research several questions need to be addressed. E.g. knowledge about the association with timing, extent of the exposure as well as lifestyle and adult SEP, is essential to address the increasing social gradient in cardiometabolic diseases.

**Methods:**

This study included a sub-sample (*N* = 264, 50% women, age 28–30) from an ongoing cohort study. We used a combination of national registers, longitudinal questionnaire data and clinical data. We examined the association between childhood SEP and cardiometabolic risk, measured by a score of multiple risk markers in young adulthood. SEP-indicators included mother’s educational level and household income. The association was evaluated by four different life course models; the latent effects model, the pathway model, the cumulative model and the social mobility model.

**Results:**

We found an inverse association between mother’s educational level and cardiometabolic risk. The association was statistically significant evaluated by the pathway and cumulative life course models, however statistically insignificant evaluated by the latent effects model. No specific association with social mobility was observed. However, high adult educational level seems to have a protecting impact on the association. No association was found between household income and cardiometabolic risk in any of the applied life course models.

**Conclusion:**

Low childhood SEP, represented by mother’s educational level but not household income, is associated with increased cardiometabolic risk in young adulthood. The accumulation of exposure, lifestyle and adult educational attainment are important for the association. In contrast, intergenerational social mobility does not seem to have a specific impact on the association and we find no evidence for a particular timing in childhood.

**Supplementary Information:**

The online version contains supplementary material available at 10.1186/s12889-022-13158-0.

## Background

Several studies have found an inverse association between socioeconomic position (SEP) in childhood and cardiometabolic diseases in adulthood [1–4]. However, many studies are cross-sectional, measuring SEP in adulthood at one time-point and assessing childhood SEP by retrospective self-reporting [4–7]. Moreover, SEP is a wide-ranging concept measured by various indicators, e.g. household income, educational attainment or occupation, with different impact and potentials for intervention. None of these indicators are stationary, and the influence of duration, timing and modifiability in the association with later health outcomes are not fully understood [8].

**Fig. 1:**
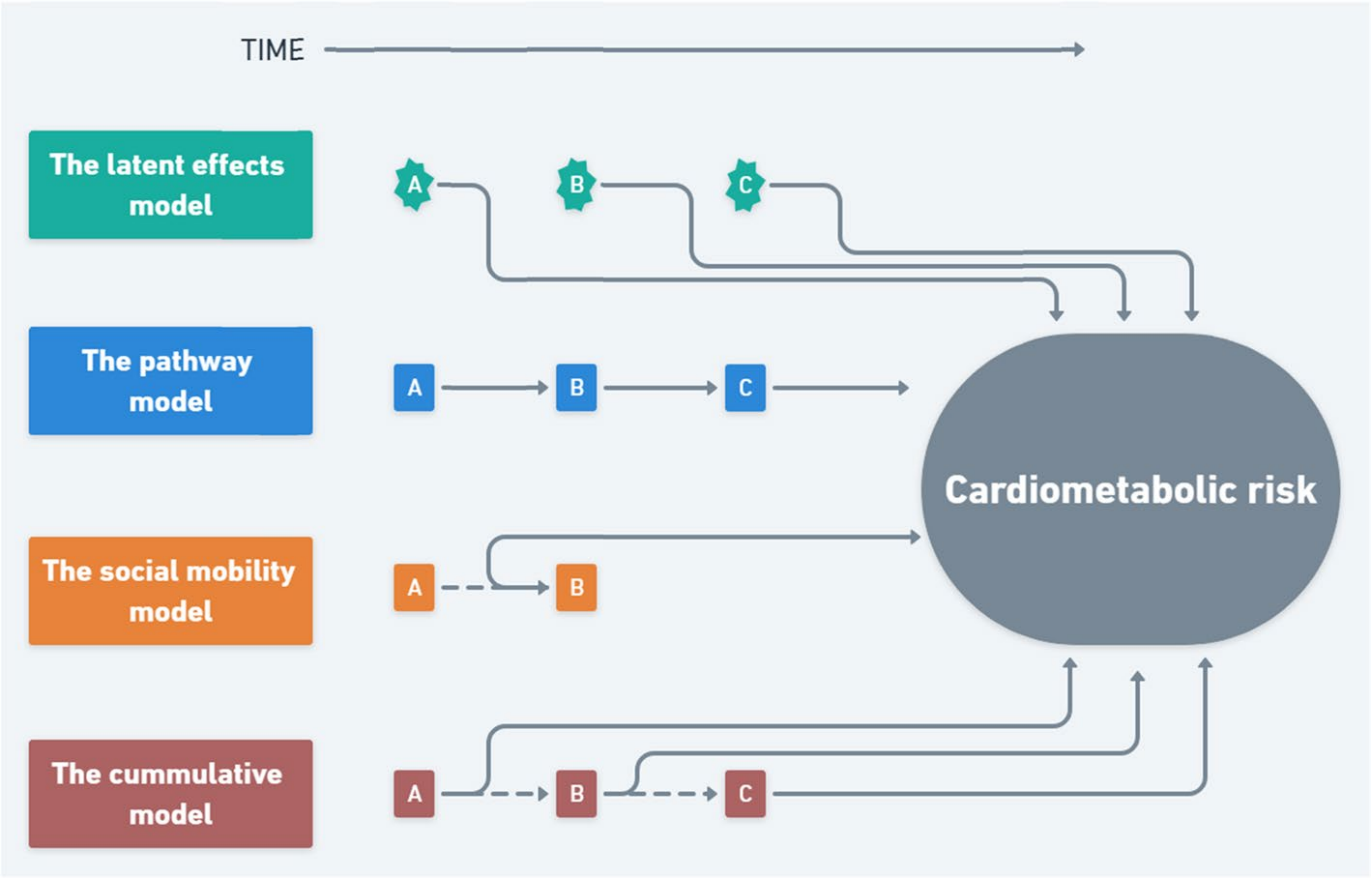
Simplified illustration of the four life course models

Four different frameworks try to capture this in life course research [9–11]. Investigating multiple life course models simultaneously utilizing the same data allows for a better comparison of how well each model describes the observed association. A simplified illustration of the four life course models are presented in Fig. 1.

*The latent effects model* evaluates certain critical/sensitive periods believed to have either irreversible or highly profound impact on the outcome of interest [12, 13]. Both early, middle and late childhood are mentioned as powerful periods due to neurobiological and social developmental processes that might influence the individual for life [14, 15].

*The pathway model* evaluates a continuity of circumstances from early life onwards. The life course is seen as a path where one life circumstance leads to the next as a trajectory of (dis) advantage [12, 16]. Not only the initial exposure but also later experiences are of interest. All elements on the path, including behavioural factors, should be included when empirically examining this model [9].

*The cumulative model* evaluates the overall accumulation of exposure across the lifespan, regardless of timing. Some researchers describe it as “health capital” that influences current and future disease risk [13, 17].

*The social mobility model* evaluates the effects of intergenerational social mobility, i.e. moving upwards or downwards on the social ladder from one position at origin to another at destination. Studies are inconsistent and four conflicting theories exist with regard to health effects of social mobility; The first suggests negative effects of any kind of mobility from increased psychological stress due to transition from one position in society to another [18]. The second suggests positive effects of upward social mobility due to a new sense of control and boosting of well-being [19]. The third suggests negative health effects of downward mobility due to the stress and feeling of unjust that emerge when accepting a new lower position [20]. The fourth is the “acculturation thesis” that focuses on the ability of mobile individuals to adapt to new environments rather than any additional effects of mobility per se [21].

The four frameworks thus focus on different consequences of the exposure to low childhood SEP: Specific timing of exposure with lasting impact independent of later experiences (evaluated by the latent effects model), the duration of the exposure independent of timing (evaluated by the cumulative model), and the later effects of the exposure (evaluated by the pathway model and social mobility model). These frameworks are often seen as competing models but prior research suggests an interdependent nature of the models and encourages the inclusion of multiple models when analysing life course perspectives in health [9, 22].

Furthermore, there is an ongoing debate regarding the best way to define measures of cardiometabolic risk in young individuals prior to manifest disease [23, 24]. Most agree that clusters of specific cardiometabolic risk markers tend to co-exist. Consequently, recent work recommends the use of multiple risk markers and continuous scales to avoid specific thresholds and to account for the interplay between different pathological domains (e.g. related to inflammation, metabolism, dyslipidaemia and thrombosis) [23–25].

In order to fill in gaps in the knowledge identified by prior research, this study was conducted to investigate the association between childhood SEP, measured prospectively by different indicators, and cardiometabolic disease risk, measured by multiple risk markers in young adulthood, within each of the four life course frameworks.

## Methods

### Study population

**Fig. 2:**
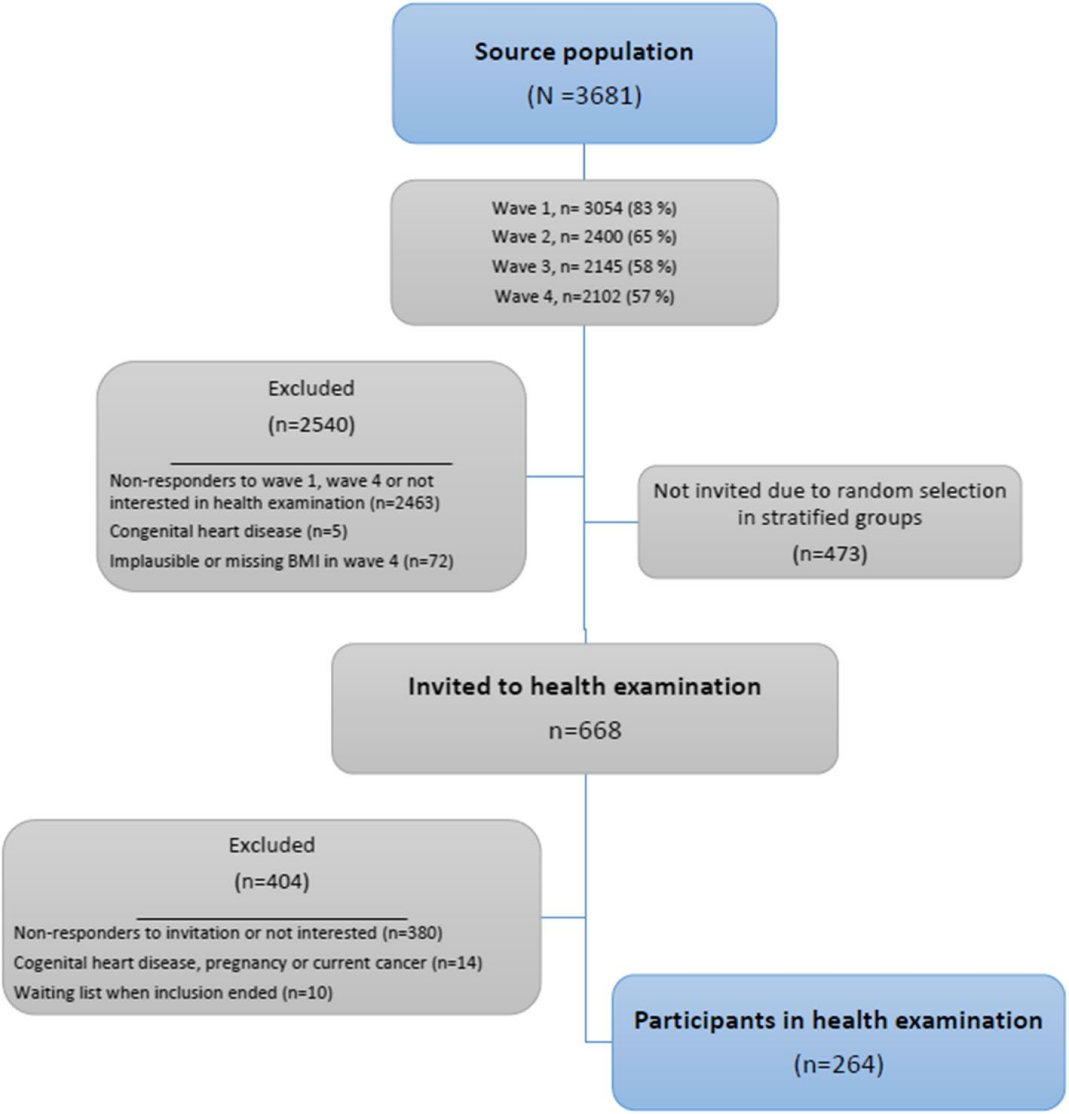
Flowchart of study population

This study included a sub-sample (*N* = 264, 50% women, age 28–30 years) from the ongoing West Jutland Cohort Study (*N* = 3681). The West Jutland Cohort consists of all individuals born in 1989, living in a specific county in Denmark in 2004. Participants were invited to fill in questionnaires at ages 15, 18, 21 and 28 years. In the latest questionnaire the participants had the opportunity to indicate interest in a health examination. If interest was indicated, and they had also filled in the initial questionnaire, they were invited into the sub-sample. They were invited based on sex and latest self-reported Body Mass Index (BMI) to obtain similar numbers in each sex- and BMI-group of individuals with normal weight, overweight and obesity (BMI < 25, 25–30 and > 30 kg/m^2^), until a total of 264 participants were included (Fig. 2). Questionnaire- and clinical data were linked to high-quality national register data from Statistics Denmark, to supplement with parental disease history, birth weight and different indicators of SEP.

### Assessment of socioeconomic position

*Childhood SEP* was evaluated by two different indicators; mother’s highest level of education and household income, representing psychosocial and material resources, respectively. Data on mother’s education was derived from educational registers and categorized into primary, secondary or tertiary education (≤10, 11–13 and > 13 years) at participant’s ages 5, 10 and 14 years [26]. Household income was defined as annual equivalised disposable income, which is a weighted scale taking the size and distribution of family members into account. This data was derived from the Income Statistics Register provided by Statistics Denmark. We averaged the mean of each year when data was available for at least 3 years in each period at participant’s ages 0–5, 6–10 and 11–15 years. We categorized the variable into low, medium and high household income at the 33.3rd and 66.6th percentiles of the entire West Jutland Cohort Study population.

*Adult SEP* was defined as participant’s highest level of education at age 28 and categorized into primary, secondary or tertiary education (≤10, 11–13 and > 13 years) using data from educational registers [26].

*SEP mobility* was defined as upward, downward or immobile when adult SEP was above, below or the same as childhood SEP.

### Definition of exposure

*The latent effects model*: SEP was assessed at age 0–5, 6–10 and 11–15 years, representing early childhood, middle childhood and late childhood as defined in previous research [22]. We evaluated all age-periods by both SEP-indicators knowing that high educational level of the mother in early childhood remains high in late childhood. However, the findings of the specific periods might be of relevance for comparison in future studies.

*The pathway model*: SEP was assessed at age 11–15 years and adult SEP at age 28 years. Lifestyle included physical activity and smoking status.

*The cumulative model*: SEP was assessed as a score summarizing the periods in the latent effects model and the participants own SEP at age 28 years. The score ranged from 0 to 8 where higher scores indicate greater exposure to low SEP. Results are presented as regression coefficients as well as categories of the level of exposure.

*The social mobility model:* Childhood SEP was assessed at age 11–15 years and adult SEP at age 28 years.

### Assessment of cardiometabolic risk

The health examinations were performed from April 2018 to December 2019 [27]. Fasting blood samples were analysed at the central laboratory, Aarhus University Hospital and supplemented with Interleukin-6 analysed at BioXpedia (Aarhus, Denmark) using Meso Scale Diagnostics Technology V-plex human pro-inflammatory panel 1.

#### Definition of outcome

The biomarkers used to define cardiometabolic risk were defined a priori and represent markers of inflammation, hypertension, glucose metabolism and lipid status and included: High-sensitive CRP, interleukin-6, fibrinogen, systolic and diastolic blood pressure, insulin, glucose, high-density lipoprotein cholesterol and triglycerides. To include potential synergistic effects of different biological domains influencing disease risk, a continuous scale of cardiometabolic risk (CMR) was constructed. A continuous scale is statistically more sensitive and less prone to error compared to dichotomous data [28].

The nine cardiometabolic biomarkers used in the CMR score were standardized (inflammatory markers on the log-scale) to eliminate risk of unequal variance, and sample-weights, represented by latest self-reported BMI-group, were applied. The standardized scores were generated for each sex separately and summarized within each biological domain. The mean values of the four domains were then summarized and standardized to create CMR. Prior to standardization, we multiplied the values of high-density lipoprotein cholesterol by − 1 to account for the inverse association with disease risk. Two participants with diabetes mellitus type 1 were excluded from the glucose metabolism domain but included in the overall CMR score.

### Assessment of additional variables

*Physical activity* was derived from questionnaires at ages 15, 18, 21 and 28. For each age-point we dichotomised the variable according to the recommended level of physical activity for Danish adolescents (1 h/day) and adults (30 min/day), respectively [29]. If the participant was missing one response this was replaced with the mean value of the three available responses. The values across all years were summarized to a scale ranging from 0 to 4, where higher scores indicate higher levels of physical activity.

*Smoking* was categorized into current, former or never smoker at age 28–30 years.

*Parental disease history* was evaluated by the participants in a questionnaire received prior to the health examination. These data were supplemented with register data from the Danish National Patient Register on cardiometabolic diagnoses from public hospitals. The diagnoses included diabetes mellitus, ischemic heart disease, acute myocardial infarction, atherosclerosis and stroke. The information was dichotomized into none or some if either of the parents had information on disease history. The variable was split into “parent with diabetes” and “parent with cardiovascular disease” depending on the specific diagnoses.

*Birth weight* was derived from the Danish Medical Birth Register that includes all national hospital- and homebirths [30]. It was categorized into high, normal and low (≥4500, 2500–4500 and < 2500 g) according to national guidelines.

### Statistical analyses

All analyses were performed with STATA software version 16.0 (STATA corporation, College Station, Texas).

Initially, descriptive statistics were performed. The distribution of CMR, the four biological domains included in CMR, parental cardiometabolic disease history and lifestyle factors were presented by SEP categories in late childhood and young adulthood as mean (standard deviation) for continuous measures and number (percentage) for categorical measures. The correlation between educational level of the mother in late childhood and adulthood educational level was evaluated by Spearman’s rank order correlation coefficient. Multiple linear regression models were fitted to estimate the association between childhood SEP and CMR by the latent effects, cumulative and pathway models as described in previous research [10]. We applied inverse probability-weights to the regression analyses to account for the sampling by BMI and sex. The models were checked by diagnostic plots of the residuals. We furthermore evaluated a potential effect measure modification of sex by including an interaction term in all models. As no significant interactions were found, all analyses were performed with both sexes together, adjusted for sex, birth weight and parental cardiometabolic disease history. When analysing social mobility, conventional linear regression models, including childhood SEP, adult SEP and mobility effects, cause potential problems due to multi-collinearity since the mobility per definition is measured by the difference between childhood and adult SEP. To take this into account we used diagonal reference models (DRM) to evaluate the distinct effects of social mobility on CMR, and further included birth weight, sex and parental disease history in the model. A detailed description of the equation used in DRM is to be found elsewhere [31]. However, DRM is specifically designed to disentangle social mobility in order to respect that outcome (measured by CMR) may be affected by both the origin (childhood SEP), destination (adult SEP) and the mobility itself [31, 32]. Furthermore, DRM estimates the relative weight of destination and origin. The measure is between 0 and 1. Hence a weight of 50% implies that origin and destination are equally important with regard to the outcome measure. Additionally, the four life course models were evaluated with respect to each of the biological domains included in the CMR score. The results concerning each distinct biological domain were presented in supplementary Tables S. 1–4.

#### Evaluating life course models

*The latent effects model:* The model suggests that latent effects from exposure to low SEP at specific periods in childhood remain, irrespective of later SEP. The model is supported if childhood SEP is inversely associated with CMR after adjustment for adult SEP at any of the three periods in childhood [10].

*The pathway model:* The model suggests indirect effects of childhood SEP through later experiences. The model is supported if childhood SEP is inversely associated with CMR prior to adjustment for lifestyle factors and adult SEP, and attenuated after this adjustment [10].

*The social mobility model:* The model suggests specific effects of either upward or downward social mobility. The model is supported if systematic differences remain in measures of CMR in social mobile individuals as compared to immobile individuals [31].

*The cumulative model:* The model suggests effects of the accumulation of exposure to low SEP throughout the life course. The model is supported if the indicators of SEP summarized throughout the life course are inversely associated with CMR [10]. The model is evaluated by a sum score of socioeconomic position in childhood (early, middle, late) and adulthood (age 28 years).

## Results

Descriptive statistics are presented in Table 1. As illustrated, a total of 264 individuals (aged 28–30 years, 50% women) participated in the health examination. There were no statistical significant differences with regard to participant’s lifestyle in the SEP-stratified groups. However, more from high childhood SEP were currently non-smokers and more often attained the recommended level of physical activity as compared to those from low SEP. The mean levels of CMR were higher in the groups with low childhood or adulthood SEP as compared to the groups with high SEP. Investigating the four biological domains separately, there was an inverse association between each domain and adult SEP, however, only the inflammatory domain was statistically significant inversely associated with childhood SEP, whereas the remainders showed minor differences across childhood SEP strata. Correlations between educational level of the mother in late childhood and adulthood educational level were rather weak with a Spearman’s rho of 0.22.

**Table 1:** Distribution of participants, cardiometabolic risk and additional variables by mother’s and own educational level

	N	Mother’s educational level, late childhood			N	Participant educational level, age 28		
		High	Average	Low		High	Average	Low
**Total, participants**	*259*	81 (31%)	115 (45%)	63 (24%)	*264*	164 (62%)	78 (30%)	22 (8%)
Men	*130*	50 (38%)	52 (40%)	28 (22%)	*132*	72 (54%)	47 (36%)	13 (10%)
Women	*129*	31 (24%)	63 (49%)	35 (27%)	*132*	92 (70%)	31 (23%)	9 (7%)
**Current smoker**		8 (10%)	21 (18%)	12 (19%)		16 (10%)	22 (28%)*	5 (23%)
**Physical activity (a)**	*234*				*236*			
0–2		54 (71%)	78 (76%)	44 (80%)		113 (73%)	52 (79%)	12 (80%)
3–4		22 (29%)	25 (24%)	11 (20%)		42 (27%)	14 (21%)	3 (20%)
**Parent with diabetes**	*259*	9 (11%)	15 (13%)	15 (24%)*	*264*	19 (12%)	18 (23%)*	3 (14%)
**Parent with cardiovascular disease**	*259*	26 (32%)	28 (24%)	23 (37%)	*264*	46 (28%)	26 (33%)	6 (27%)
**CMR (b)**	*259*	0.1 (0.9)	0.1 (1.1)	0.5 (0.9)*	*264*	0.0 (1.0)	0.5 (1.0)**	0.8 (1.2)**
**Biological domains of CMR (b)**								
Inflammation	259	0.1 (1.0)	0.1 (1.0)	0.6 (1.0)**	264	0.1 (1.0)	0.5 (1.0)*	0.5 (1.1)
Lipid status	259	0.1 (0.8)	0.1 (1.1)	0.4 (1.0)	264	0.0 (0.9)	0.5 (0.9)**	0.5 (1.5)*
Glucose metabolism	257	0.1 (1.0)	0.1 (1.2)	0.2 (0.9)	262	0.1 (1.0)	0.3 (1.2)	0.6 (1.2)*
Hypertension	259	0.1 (1.0)	0.0 (1.0)	0.2 (0.8)	264	0.0 (0.9)	0.2 (0.9)	0.7 (1.1)**

*Abbreviations*: *CMR* Cardiometabolic risk

Data are presented as mean (SD) for continuous measures, and n (%) for categorical measures. *P*-values are conducted from ANOVA for continuous measures and Pearson’s chi-squared test for categorical measures for questionnaire and clinical data. *P < 0.05 compared to “High” (bold text), **P < 0.001 compared to “High” (bold text)

^a^Number of questionnaire rounds with recommended level of physical activity

^b^Standardized values, with sample-weights applied

### Life course models

Results from the adjusted analyses of mother’s educational level and CMR evaluated by each of the four life course models are presented below and in Table 2. Crude estimates are presented in supplementary Table S. 5.

**Table 2:** The association between mother’s educational level and cardiometabolic risk in young adulthood evaluated by four life course models

	Adjusted cardiometabolic risk score (95% confidence interval)^a^				
	N		Mother's highest educational level		
			High	Average	Low
**The latent effects model**					
Early childhood	*246*		**Base level**	−0.2 (− 0.5;0.1)	0.2 (− 0.1;0.6)
Middle childhood	*246*		**Base level**	−0.2 (− 0.5;0.1)	0.3 (− 0.1;0.6)
**Late childhood**	*249*		**Base level**	−0.2 (− 0.5;0.1)	0.3 (− 0.1;0.6)
**The pathway model**					
Prior to adjustment for lifestyle and adult SEP	*249*		**Base level**	−0.1 (− 0.4;0.2)	**0.4 (0.1;0.7)**
After adjustment for lifestyle and adult SEP	*227*		**Base level**	−0.2 (− 0.5;0.1)	0.3 (− 0.1;0.6)
**The social mobility model**	*249*				
Adult educational level: High			−0.1 (− 0.9;0.7)	0.0 (− 0.8;0.8)	0.0 (− 0.8;0.9)
Adult educational level: Average			0.3 (−0.6;1.1)	0.3 (−0.5;1.1)	0.4 (− 0.4;1.2)
Adult educational level: Low			0.7 (− 0.2;1.5)	0.7 (− 0.1;1.6)	0.8 (− 0.1;1.8)
Separate upward mobility coefficient		−0.3 (− 0.8;0.2)			
Separate downward mobility coefficient		−0.1 (− 0.6;0.5)			
** The cumulative model**	*246*				
Regression coefficient		**0.1 (0.0;0.1)**			
0–2		**Base level**			
3–5		−0.1 (−0.4;0.2)			
6–8		**0.5 (0.1;0.8)**			

*SEP* Socioeconomic position

^a^Adjusted for sex, birth weight and parental cardiometabolic diseases

#### The latent effects model

Evaluating the association between childhood SEP and CMR by the latent effects model, no statistically significant differences between those growing up in families with high SEP and those growing up in families with average or low SEP were observed. However, there was a tendency towards increased levels of CMR among those in the low SEP stratum compared to those in the average or high SEP strata. The results were similar in early, middle and late childhood.

#### The pathway model

Evaluating the association between childhood SEP and CMR by the pathway model, we found statistically significant increased CMR among those growing up in families with low SEP compared to those with average or high SEP. The estimates were attenuated after adjustment for lifestyle and adult SEP, thus supporting the pathway model.

#### The social mobility model

Evaluating the association between intergenerational social mobility and CMR by DRM, we found the weight of destination to be greater than that of origin (74% vs. 26%, standard error 0.15).

We found no separate association with neither upwards or downwards mobility and CMR.

#### The cumulative model

Evaluating the association between accumulated exposure to low SEP and CMR, we found the greatest mean CMR among those with the greatest exposure to low SEP. This association remained statistically significant in the adjusted analysis.

Evaluating the association between household income and CMR, we found no associations in any of the adjusted life course models (Table 3).

**Table 3:** The association between household income in childhood and cardiometabolic risk in young adulthood evaluated by four life course models

	N	Adjusted cardiometabolic risk score (95% confidence interval)^a^			
			Household income		
			High	Average	Low
**The latent effects model**					
Early childhood	*252*		**Base level**	−0.1 (−0.4;0.3)	0.0 (− 0.3;0.3)
Middle childhood	*251*		**Base level**	−0.2 (− 0.5;0.1)	−0.1 (− 0.4;0.3)
Late childhood	*249*		**Base level**	0.1 (− 0.2;0.4)	− 0.1 (− 0.5;0.2)
**The pathway model**					
Prior to adjustment for lifestyle and adult SEP	*249*		**Base level**	0.0 (−0.3;0.3)	− 0.1 (− 0.5;0.3)
After adjustment for lifestyle and adult SEP	*224*		**Base level**	0.0 (−0.3;0.3)	−0.3 (− 0.7;0.1)
**The social mobility model**	*249*				
Adult educational level: High			0.0 (−0.9;0.8)	−0.1 (− 0.9;0.7)	−0.1 (− 0.9;0.7)
Adult educational level: Average			0.4 (−0.4;1.2)	0.4 (−0.4;1.2)	0.4 (− 0.4;1.2)
Adult educational level: Low			0.8 (−0.2;1.7)	0.7 (−0.2;1.6)	0.7 (− 0.2;1.6)
Separate upward mobility coefficient		−0.5 (−1.2;0.1)			
Separate downward mobility coefficient		−0.2 (− 0.9;0.5)			
**The cumulative model**	*248*				
Regression coefficient		0.0 (0.0;0.1)			
0–2		**Base level**			
3–5		0.1 (−0.2;0.4)			
6–8		0.0 (− 0.4;0.4)			

*SEP* Socioeconomic position

^a^Adjusted for sex, birth weight and parental cardiometabolic diseases

## Discussion

The main finding of this study was that children growing up in families with low SEP, measured by mother’s highest level of education, are at greater risk of developing cardiometabolic diseases later in life, evaluated by a scale of cardiometabolic risk markers at ages 28–30 years. Concerning different life course models evaluating the association, we found support for the pathway and the cumulative life course models. Moreover, we found a statistically insignificant tendency towards increased cardiometabolic risk among those from low SEP evaluated by the latent effects model. This tendency was independent of the timing of the exposure in childhood. We found no separate association with intergenerational social mobility. However, we found basis for a protective effect of higher adult SEP, represented by educational attainment at age 28 years.

The current study is not the first to address the association between childhood SEP and later cardiometabolic risk by different life course models. Our findings are in line with former research showing that accumulated SEP across the life span is the best fitting life course model concerning adult health [9, 33]. However, most of the studies do not evaluate the pathway model. This was however evaluated by a study from the 1958 British Birth Cohort [34]. They found an inverse association between childhood SEP and allostatic load at age 44. They furthermore demonstrated that the most important indirect pathway was through participants own educational attainment followed by lifestyle factors.

Evaluating intergenerational social mobility, our study did not find a separate association with adult cardiometabolic health. However, we found the association with adult SEP to be greater than childhood SEP. These findings are in line with the acculturation thesis, stating that mobile individuals absorb their new surroundings and thus have greater impact from the destination than the origin [21]. A recent study by Savitsky et al. investigated social mobility by self-reported parental and adult occupation and education (*N* = 1132) [35]. Outcome measures included anthropometry and traditional risk markers at age 32. The study pointed to adverse cardiometabolic outcome among downward and (mainly) upward mobile individuals. This was in contrast to the findings of the current study and displays the inconsistency within the social mobility literature. As opposed to the outcome measure of the current study Savitsky et al. did not include any markers of inflammation. Furthermore, the study used linear regression models to investigate the associations as opposed to the DRM used in the current study which might partly explain the different findings.

Some inconsistency does exist with regard to evaluating the association between childhood SEP and later cardiometabolic diseases in a life course perspective. Some of this inconsistency rely on different interpretations of the life course, i.e. different life course models and different interpretations of each model, and furthermore the use of different SEP-indicators. For instance, one approach is to evaluate the association after adjustment for traditional confounders (e.g. smoking and physical activity), thus neglecting to see these factors as downstream effects of childhood SEP as suggested by the pathway model [9]. Evaluating the association between childhood SEP and cardiometabolic health by another SEP indicator, household income, we found no association regardless of the applied life course model. This is of interest as the two SEP indicators represent different aspects of SEP. The former representing psychosocial aspects and the latter material resources [8]. In contrast to the findings of the current study a very recent study by Najmal et al. investigated the association between family poverty (income) and traditional cardiometabolic risk markers in young adulthood (*N* = 1297) [36]. They found statistically significant increased risk for women with family poverty as compared to those without. They found no association for men. The negative findings concerning income in the current study might be explained by the study context in a Danish welfare society with a high degree of social security. However, the link between educational level and cardiometabolic health remains largely unexplained. Is educational attainment protective due to better cognitive skills, greater knowledge and increased awareness about e.g. healthy lifestyle and public preventive strategies, also known as health literacy [37, 38]? Or is educational level also an indicator of other factors in childhood that influence both cardiometabolic health and educational attainment such as network, stress, parenting styles etc. [39]? Our descriptive results revealed increased inflammatory markers in those growing up in families with low SEP. Growing evidence suggests an association between various psychosocial stressors and low-grade inflammation [6, 40, 41]. This could indicate a potential link that needs attention in future research.

### Strengths and limitations

Some limitations need to be addressed. Since this study is based on a sub-sample of a youth cohort, attrition and selection might bias the results. We applied probability weights in all regression-analyses and to the outcome measure to account for the sampling by sex and BMI-group. Respondents to the questionnaires and participants in the health examination had higher SEP as compared to the source population [27, 42]. Unfortunately, it is not possible to know whether this selection was associated with cardiometabolic health and thus inducing differential selection bias. Previous research indicates that participation in studies is more likely with better health [43]. If this is the case in our study, the selection might have attenuated the results. The association between childhood SEP and cardiometabolic risk was investigated by a score of multiple biomarkers in a population of young adults prior to the development of manifest diseases. It is uncertain to what degree this score translates into clinical diseases. However, all biomarkers included in the score were known risk markers of cardiometabolic diseases and the approach has been used in a similar manner in previous studies [44–46]. Testing multiple life course models simultaneously might introduce the risk of false-positive conclusions (Type 1 error). Since all of the life course models were pre-established hypothesis we decided to avoid the risk of false-negative conclusions (Type 2 error) which could be introduced by applying a more restrictive approach of e.g. multiple hypothesis adjustment of the results [47]. However, adding Bonferroni correction did not change any of the overall conclusions.

The main strength of our study is the use of high quality registers in combination with longitudinal questionnaire information and a comprehensive panel of clinical biomarkers. This facilitated the empirical exploration of childhood SEP and cardiometabolic risk by all four life course models in addition to different SEP-indicators.

We used continuous scales of interrelated cardiometabolic risk markers rather than arbitrary cut-off values. This was done to respect the potential of synergistic effects of different biological domains influencing disease risk. Furthermore, investigating the impact of SEP prior to manifest diseases has the advantage of reducing potential epidemiological challenges due to bias from differences across SEP in relation to e.g. health care utility, healthcare provider bias and adherence to treatment [45]. Furthermore, some of the inconsistency in the social mobility literature might be explained by methodological and analytical challenges and the use of DRM is seen as a strength of this study [31].

## Conclusion

In conclusion, this study strengthens the evidence for an overall association between the educational level of the mother and cardiometabolic risk in young adulthood. We found empirical support for the cumulative and pathway life course models. We found no specific timing across three different periods in childhood and no specific association with intergenerational social mobility. These findings emphasize the need to understand the underlying pathophysiological mechanisms as dynamic in nature and that improved cardiometabolic health can be gained throughout different developmental periods increasing the possibility for interventions. Improved understanding of the association with regard to health literacy, psychosocial stressors and dysregulated physiology is critical to inform policy makers and improve cardiometabolic prevention to support the continuing health of all children.

## Supplementary Information

**Additional file 1.**

## Data Availability

Restrictions apply to the availability of some or all data generated or analysed during this study to preserve patient confidentiality or because they were used under license. The corresponding author will on request detail the restrictions and any conditions under which access to some data may be provided.

## Abbreviations

BMI: Body Mass Index
CMR: Cardiometabolic Risk
DRM: Diagonal Reference Models
SEP: Socioeconomic Position

## References

[1] Power C, Atherton K, Strachan DP, Shepherd P, Fuller E, Davis A (2007). Life-course influences on health in British adults: effects of socio-economic position in childhood and adulthood. Int J Epidemiol.

[2] Blane D, Hart CL, Smith GD, Gillis CR, Hole DJ, Hawthorne VM (1996). Association of cardiovascular disease risk factors with socioeconomic position during childhood and during adulthood. BMJ.

[3] Hostinar CE, Ross KM, Chen E, Miller GE (2017). Early-life socioeconomic disadvantage and metabolic health disparities. Psychosom Med.

[4] Galobardes B, Smith GD, Lynch JW (2006). Systematic review of the influence of childhood socioeconomic circumstances on risk for cardiovascular disease in adulthood. Ann Epidemiol.

[5] Matthews KA, Gallo LC (2011). Psychological perspectives on pathways linking socioeconomic status and physical health. Annu Rev Psychol.

[6] Milaniak I, Jaffee SR (2019). Childhood socioeconomic status and inflammation: a systematic review and meta-analysis. Brain Behav Immun.

[7] Slopen N, Goodman E, Koenen KC, Kubzansky LD (2013). Socioeconomic and other social stressors and biomarkers of cardiometabolic risk in youth: a systematic review of less studied risk factors. PLoS One.

[8] Galobardes B, Shaw M, Lawlor DA, Lynch JW, Davey SG (2006). Indicators of socioeconomic position (part 1). J Epidemiol Community Health.

[9] Pollitt RA, Rose KM, Kaufman JS (2005). Evaluating the evidence for models of life course socioeconomic factors and cardiovascular outcomes: a systematic review. BMC Public Health.

[10] Walsemann KM, Goosby BJ, Farr D (2016). Life course SES and cardiovascular risk: heterogeneity across race/ethnicity and gender. Soc Sci Med.

[11] Green MJ, Popham F (2017). Life course models: improving interpretation by consideration of total effects. Int J Epidemiol.

[12] Hertzman C, Power C, Matthews S, Manor O (2001). Using an interactive framework of society and lifecourse to explain self-rated health in early adulthood. Soc Sci Med.

[13] Kuh D, Shlomo YB (1997). A life course approach to chronic disease epidemiology: tracing the origins of ill health from early to adult life.

[14] Kuhlman KR, Chiang JJ, Horn S, Bower JE (2017). Developmental psychoneuroendocrine and psychoneuroimmune pathways from childhood adversity to disease. Neurosci Biobehav Rev.

[15] National Research Council (US) Panel to Review the Status of Basic Research on School-Age Children, Collins WA, eds. Development During Middle Childhood: The Years From Six to Twelve. Washington (DC): National Academies Press (US); 1984. 25032422

[16] Power C, Hertzman C (1997). Social and biological pathways linking early life and adult disease. Br Med Bull.

[17] Pollitt RA, Kaufman JS, Rose KM, Diez-Roux AV, Zeng D, Heiss G (2008). Cumulative life course and adult socioeconomic status and markers of inflammation in adulthood. J Epidemiol Community Health.

[18] Sorokin PA (1959). Social and cultural mobility.

[19] Gugushvili A, Zhao Y, Bukodi E (2019). ‘Falling from grace’ and ‘rising from rags’: intergenerational educational mobility and depressive symptoms. Soc Sci Med.

[20] Newman KS (1988). Falling from grace: downward mobility in the age of affluence.

[21] Blau PM (1956). Social mobility and interpersonal relations. Am Sociol Rev.

[22] Green MJ, Stritzel H, Smith C, Popham F, Crosnoe R (2018). Timing of poverty in childhood and adolescent health: evidence from the US and UK. Soc Sci Med.

[23] Huang RC, Mori TA, Burke V, Newnham J, Stanley FJ, Landau LI (2009). Synergy between adiposity, insulin resistance, metabolic risk factors, and inflammation in adolescents. Diabetes Care.

[24] Huang RC, Prescott SL, Godfrey KM, Davis EA (2015). Assessment of cardiometabolic risk in children in population studies: underpinning developmental origins of health and disease mother-offspring cohort studies. J Nutr Sci.

[25] Hoogeveen RM, Pereira JPB, Nurmohamed NS, Zampoleri V, Bom MJ, Baragetti A (2020). Improved cardiovascular risk prediction using targeted plasma proteomics in primary prevention. Eur Heart J.

[26] Jensen VM, Rasmussen AW (2011). Danish education registers. Scand J Public Health.

[27] Kempel MK, Winding TN, Lynggaard V, Brantlov S, Andersen JH, Böttcher M (2021). Traditional and novel cardiometabolic risk markers across strata of body mass index in young adults. Obes Sci Pract.

[28] Ragland DR (1992). Dichotomizing continuous outcome variables: dependence of the magnitude of association and statistical power on the cutpoint. Epidemiology.

[29] Pedersen BKAL (2011). Fysisk aktivitet – håndbog om forebyggelse og behandling.

[30] Bliddal M, Broe A, Pottegård A, Olsen J, Langhoff-Roos J (2018). The Danish medical birth register. Eur J Epidemiol.

[31] van der Waal J, Daenekindt S, de Koster W (2017). Statistical challenges in modelling the health consequences of social mobility: the need for diagonal reference models. Int J Public Health..

[32] Sobel M (1981). Diagonal mobility models: a substantively motivated class of designs for the analysis of mobility effects. Am Sociol Rev.

[33] Robertson T, Popham F, Benzeval M (2014). Socioeconomic position across the lifecourse & allostatic load: data from the west of Scotland Twenty-07 cohort study. BMC Public Health.

[34] Barboza Solís C, Fantin R, Castagné R, Lang T, Delpierre C, Kelly-Irving M (2016). Mediating pathways between parental socio-economic position and allostatic load in mid-life: findings from the 1958 British birth cohort. Soc Sci Med.

[35] Savitsky B, Manor O, Friedlander Y, Burger A, Lawrence G, Calderon-Margalit R (2017). Associations of socioeconomic position in childhood and young adulthood with cardiometabolic risk factors: the Jerusalem perinatal family follow-up study. J Epidemiol Community Health.

[36] Najman JM, Wang W, Plotnikova M, Mamun AA, McIntyre D, Williams GM (2020). Poverty over the early life course and young adult cardio-metabolic risk. Int J Public Health.

[37] Friis K, Lasgaard M, Rowlands G, Osborne RH, Maindal HT (2016). Health literacy mediates the relationship between educational attainment and health behavior: a Danish population-based study. J Health Commun.

[38] Laaksonen M, Talala K, Martelin T, Rahkonen O, Roos E, Helakorpi S (2008). Health behaviours as explanations for educational level differences in cardiovascular and all-cause mortality: a follow-up of 60 000 men and women over 23 years. Eur J Pub Health.

[39] Hemmingsson E (2018). Early childhood obesity risk factors: socioeconomic adversity, family dysfunction, offspring distress, and junk food self-medication. Curr Obes Rep.

[40] Schmeer KK, Yoon A (2016). Socioeconomic status inequalities in low-grade inflammation during childhood. Arch Dis Child.

[41] Su S, Jimenez MP, Roberts CT, Loucks EB (2015). The role of adverse childhood experiences in cardiovascular disease risk: a review with emphasis on plausible mechanisms. Curr Cardiol Rep.

[42] Winding TN, Andersen JH, Labriola M, Nohr EA (2014). Initial non-participation and loss to follow-up in a Danish youth cohort: implications for relative risk estimates. J Epidemiol Community Health.

[43] Greenberg RSDS, Flanders W, Eley JW, Boring JR (2005). Medical Epidemiology.

[44] Non AL, Rewak M, Kawachi I, Gilman SE, Loucks EB, Appleton AA (2014). Childhood social disadvantage, cardiometabolic risk, and chronic disease in adulthood. Am J Epidemiol.

[45] Winning A, Glymour MM, McCormick MC, Gilsanz P, Kubzansky LD (2015). Psychological distress across the life course and Cardiometabolic risk: findings from the 1958 British birth cohort study. J Am Coll Cardiol.

[46] Kamel M, Smith BT, Wahi G, Carsley S, Birken CS, Anderson LN (2018). Continuous cardiometabolic risk score definitions in early childhood: a scoping review. Obes Rev.

[47] Armstrong RA (2014). When to use the Bonferroni correction. Ophthalmic Physiol Opt.

