# Supplementary material for: Evaluating the association between socioeconomic position and cardiometabolic risk markers in young adulthood by different life course models
Source: BMC Public Health. 2022 Apr 9;22:694. doi: 10.1186/s12889-022-13158-0 (PMC8994289; doi:10.1186/s12889-022-13158-0)
Supplement: Supplementary file 1 — Additional file 1. [file 12889_2022_13158_MOESM1_ESM.docx]

Table S. 1 The association between mother's educational level and the **inflammatory domain** risk score evaluated by four life course models

|  | Adjusted inflammatory risk score (95 % confidence interval)* | | | | | | | |
| --- | --- | --- | --- | --- | --- | --- | --- | --- |
|  |  | | |  | **Mothers highest educational level** | | | |
|  | **N** | |  | | **High** | **Average** | | **Low** |
| The latent effects model |  |  | | |  |  |  | |
| Early childhood | *246* |  | | | **Base level** | -0.1 (-0.4;0.2) | 0.3 (0.0;0.7) | |
| Middle childhood | *246* |  | | | **Base level** | -0.1 (-0.4;0.2) | 0.4 (0.0;0.7) | |
| Late childhood | *249* |  | | | **Base level** | 0.0 (-0.3;0.3) | **0.4 (0.0;0.8)** | |
|  |  |  | | |  |  |  | |
| The pathway model |  |  | | |  |  |  | |
| Prior to adjustment for lifestyle and adult SEP | *249* |  | | | **Base level** | 0.0 (-0.3;0.4) | **0.5 (0.1;0.9)** | |
| After adjustment for lifestyle and adult SEP | *227* |  | | | **Base level** | 0.0 (-0.3;0.3) | **0.4 (0.0;0.8)** | |
|  |  |  | | |  |  |  | |
| The social mobility model | *249* |  | | |  |  |  | |
| Adult educational level: High |  |  | | | -0.4 (-1.2;0.4) | -0.3 (-1.0;0.5) | 0.1 (-0.7;1.0) | |
| Adult educational level: Average |  |  | | | -0.3 (-1.1;0.5) | -0.2 (-1.0;0.6) | 0.2 (-0.6;1.0) | |
| Adult educational level: Low |  |  | | | -0.1 (-1.0;0.8) | 0.0 (-0.9;0.9) | 0.4 (-0.5;1.3) | |
|  |  |  | | |  |  |  | |
| Separate upward mobility coefficient |  | -0.4 (-0.8;0.0) | | |  |  |  | |
| Separate downward mobility coefficient |  | -0.4 (-0.9;0.2) | | |  |  |  | |
|  |  |  | | |  |  |  | |
| The cumulative model | *246* |  | | |  |  |  | |
| Regression coefficient |  | **0.1 (0.0;0.1)** | | |  |  |  | |
|  |  |  | | |  |  |  | |
| 0-2 |  | **Base level** | | |  |  |  | |
| 3-5 |  | 0.1 (-0.3;0.4) | | |  |  |  | |
| 6-8 |  | **0.5 (0.1;0.9)** | | |  |  |  | |

- The latent effects model: Evaluated by a potential latent effect of timing across three periods in childhood. Adjusted for adult SEP.
- The pathway model: Evaluated by a potential indirect effect of downstream factors by comparing the estimates prior and after adjustment for lifestyle and adult SEP.
- The social mobility model: Evaluated by separate effects of upward and downward intergenerational mobility.
- The cumulative model: Evaluated by a sum score of socioeconomic position in childhood (early, middle, late) and adulthood (age 28 years). The score is ranging from 0-8 and higher scores indicate greater exposure to low SEP. Results are presented as regression coefficients as well as categories of the level of exposure.

*Adjusted for sex, birthweight and parental cardiometabolic diseases. SEP, socioeconomic position

Table S. 2 The association between mother's educational level and the **lipid domain** risk score evaluated by four life course models

|  | Adjusted lipid risk score (95 % confidence interval)* | | | | | | | |
| --- | --- | --- | --- | --- | --- | --- | --- | --- |
|  |  | | |  | **Mothers highest educational level** | | | |
|  | **N** | |  | | **High** | **Average** | | **Low** |
| The latent effects model |  |  | | |  |  |  | |
| Early childhood | *246* |  | | | **Base level** | 0.0 (-0.4;0.3) | 0.2 (-0.2;0.5) | |
| Middle childhood | *246* |  | | | **Base level** | -0.1 (-0.4;0.2) | 0.3 (-0.1;0.6) | |
| Late childhood | *249* |  | | | **Base level** | -0.1 (-0.4;0.2) | 0.3 (-0.1;0.6) | |
|  |  |  | | |  |  |  | |
| The pathway model |  |  | | |  |  |  | |
| Prior to adjustment for lifestyle and adult SEP | *249* |  | | | **Base level** | 0.0 (-0.3;0.3) | **0.4 (0.0;0.8)** | |
| After adjustment for lifestyle and adult SEP | *227* |  | | | **Base level** | -0.1 (-0.4;0.2) | 0.3 (-0.2;1.2) | |
|  |  |  | | |  |  |  | |
| The social mobility model | *249* |  | | |  |  |  | |
| Adult educational level: High |  |  | | | 0.0 (-0.8;0.8) | 0.0 (-0.8;0.8) | 0.0 (-0.8;0.8) | |
| Adult educational level: Average |  |  | | | 0.5 (-0.3;1.3) | 0.4 (-0.3;1.2) | 0.4 (-0.3;1.2) | |
| Adult educational level: Low |  |  | | | 0.5 (-0.4;1.4) | 0.5 (-0.4;1.4) | 0.5 (-0.4;1.4) | |
|  |  |  | | |  |  |  | |
| Separate upward mobility coefficient |  | 0.2 (-0.3;0.7) | | |  |  |  | |
| Separate downward mobility coefficient |  | -0.2 (-0.8;0.3) | | |  |  |  | |
|  |  |  | | |  |  |  | |
| The cumulative model | *246* |  | | |  |  |  | |
| Regression coefficient |  | **0.1 (0.0;0.1)** | | |  |  |  | |
|  |  |  | | |  |  |  | |
| 0-2 |  | **Base level** | | |  |  |  | |
| 3-5 |  | 0.0 (-0.3;0.3) | | |  |  |  | |
| 6-8 |  | **0.4 (0.1;0.8)** | | |  |  |  | |

- The latent effects model: Evaluated by a potential latent effect of timing across three periods in childhood. Adjusted for adult SEP.
- The pathway model: Evaluated by a potential indirect effect of downstream factors by comparing the estimates prior and after adjustment for lifestyle and adult SEP.
- The social mobility model: Evaluated by separate effects of upward and downward intergenerational mobility.
- The cumulative model: Evaluated by a sum score of socioeconomic position in childhood (early, middle, late) and adulthood (age 28 years). The score is ranging from 0-8 and higher scores indicate greater exposure to low SEP. Results are presented as regression coefficients as well as categories of the level of exposure.

*Adjusted for sex, birthweight and parental cardiometabolic diseases. SEP, socioeconomic position

Table S. 3 The association between mother's educational level and the **glucose-metabolism domain** risk score evaluated by four life course models

|  | Adjusted glucose-metabolism risk score (95 % confidence interval)* | | | | | | | |
| --- | --- | --- | --- | --- | --- | --- | --- | --- |
|  |  | | |  | **Mothers highest educational level** | | | |
|  | **N** | |  | | **High** | **Average** | | **Low** |
| The latent effects model |  |  | | |  |  |  | |
| Early childhood | *244* |  | | | **Base level** | -0.1 (-0.5;0.2) | 0.1 (-0.2;0.5) | |
| Middle childhood | *244* |  | | | **Base level** | -0.2 (-0.5;0.1) | 0.1 (-0.2;0.5) | |
| Late childhood | *247* |  | | | **Base level** | -0.2 (-0.5;0.1) | 0.1 (-0.3;0.4) | |
|  |  |  | | |  |  |  | |
| The pathway model |  |  | | |  |  |  | |
| Prior to adjustment for lifestyle and adult SEP | *247* |  | | | **Base level** | -0.1 (-0.4;0.2) | 0.2 (-0.2;0.5) | |
| After adjustment for lifestyle and adult SEP | *225* |  | | | **Base level** | -0.2 (-0.5;0.1) | 0.1 (-0.3;0.4) | |
|  |  |  | | |  |  |  | |
| The social mobility model | *247* |  | | |  |  |  | |
| Adult educational level: High |  |  | | | -0.2 (-1.1;0.7) | -0.2 (-1.1;0.7) | -0.2 (-1.2;0.7) | |
| Adult educational level: Average |  |  | | | 0.0 (-0.9;0.9) | 0.0 (-0.9;0.9) | 0.0 (-0.9;0.9) | |
| Adult educational level: Low |  |  | | | 0.3 (-0.7;1.4) | 0.3 (-0.7;1.3) | 0.3 (-0.7;1.3) | |
|  |  |  | | |  |  |  | |
| Separate upward mobility coefficient |  | -0.4 (-1.2;0.4) | | |  |  |  | |
| Separate downward mobility coefficient |  | 0.1 (-0.6;0.8) | | |  |  |  | |
|  |  |  | | |  |  |  | |
| The cumulative model | *244* |  | | |  |  |  | |
| Regression coefficient |  | 0.0 (0.0;0.1) | | |  |  |  | |
|  |  |  | | |  |  |  | |
| 0-2 |  | **Base level** | | |  |  |  | |
| 3-5 |  | -0.1 (-0.4;0.2) | | |  |  |  | |
| 6-8 |  | 0.3 (-0.01;0.6) | | |  |  |  | |

- The latent effects model: Evaluated by a potential latent effect of timing across three periods in childhood. Adjusted for adult SEP.
- The pathway model: Evaluated by a potential indirect effect of downstream factors by comparing the estimates prior and after adjustment for lifestyle and adult SEP.
- The social mobility model: Evaluated by separate effects of upward and downward intergenerational mobility.
- The cumulative model: Evaluated by a sum score of socioeconomic position in childhood (early, middle, late) and adulthood (age 28 years). The score is ranging from 0-8 and higher scores indicate greater exposure to low SEP. Results are presented as regression coefficients as well as categories of the level of exposure.

*Adjusted for sex, birthweight and parental cardiometabolic diseases. SEP, socioeconomic position

Table S. 4 The association between mother's educational level and the **hypertension domain** risk score evaluated by four life course models

|  | Adjusted hypertension risk score (95 % confidence interval)* | | | | | | | |
| --- | --- | --- | --- | --- | --- | --- | --- | --- |
|  |  | | |  | **Mothers highest educational level** | | | |
|  | **N** | |  | | **High** | **Average** | | **Low** |
| The latent effects model |  |  | | |  |  |  | |
| Early childhood | *246* |  | | | **Base level** | -0.2 (-0.6;0.2) | 0.0 (-0.3;0.3) | |
| Middle childhood | *246* |  | | | **Base level** | -0.2 (-0.5;0.2) | 0.0 (-0.3;0.4) | |
| Late childhood | *249* |  | | | **Base level** | -0.3 (-0.6;0.1) | 0.0 (-0.4;0.3) | |
|  |  |  | | |  |  |  | |
| The pathway model |  |  | | |  |  |  | |
| Prior to adjustment for lifestyle and adult SEP | *249* |  | | | **Base level** | -0.2 (-0.6;0.1) | 0.0 (-0.3;0.3) | |
| After adjustment for lifestyle and adult SEP | *227* |  | | | **Base level** | -0.2 (-0.6;0.2) | 0.0 (-0.4;0.4) | |
|  |  |  | | |  |  |  | |
| The social mobility model | *249* |  | | |  |  |  | |
| Adult educational level: High |  |  | | | 0.3 (-0.5;1.1) | 0.3 (-0.5;1.1) | 0.3 (-0.5;1.2) | |
| Adult educational level: Average |  |  | | | 0.5 (-0.3;1.3) | 0.5 (-0.2;1.3) | 0.6 (-0.2;1.4) | |
| Adult educational level: Low |  |  | | | 1.0 (0.2;1.9) | 1.1 (0.2;1.9) | 1.1 (0.2;2.0) | |
|  |  |  | | |  |  |  | |
| Separate upward mobility coefficient |  | -0.1 (-0.5;0.3) | | |  |  |  | |
| Separate downward mobility coefficient |  | 0.2 (-0.4;0.7) | | |  |  |  | |
|  |  |  | | |  |  |  | |
| The cumulative model | *246* |  | | |  |  |  | |
| Regression coefficient |  | 0.0 (0.0;0.1) | | |  |  |  | |
|  |  |  | | |  |  |  | |
| 0-2 |  | **Base level** | | |  |  |  | |
| 3-5 |  | -0.2 (-0.6;0.2) | | |  |  |  | |
| 6-8 |  | 0.1 (-0.3;0.4) | | |  |  |  | |

- The latent effects model: Evaluated by a potential latent effect of timing across three periods in childhood. Adjusted for adult SEP.
- The pathway model: Evaluated by a potential indirect effect of downstream factors by comparing the estimates prior and after adjustment for lifestyle and adult SEP.
- The social mobility model: Evaluated by separate effects of upward and downward intergenerational mobility.
- The cumulative model: Evaluated by a sum score of socioeconomic position in childhood (early, middle, late) and adulthood (age 28 years). The score is ranging from 0-8 and higher scores indicate greater exposure to low SEP. Results are presented as regression coefficients as well as categories of the level of exposure.

*Adjusted for sex, birthweight and parental cardiometabolic diseases. SEP, socioeconomic position

Table S. 5. The crude association between mother's educational level and cardiometabolic risk score evaluated by four life course models

|  |  | Crude cardiometabolic risk score (95 % confidence interval) | | | |
| --- | --- | --- | --- | --- | --- |
|  |  |  | Mothers highest educational level | | |
|  | N |  | **High** | **Average** | **Low** |
| The latent effects model |  |  |  |  |  |
| Early childhood | *248* |  | **Base level** | -0.2 (-0.5;0.1) | 0.3 (-0.1;0.6) |
| Middle childhood | *255* |  | **Base level** | -0.2 (-0.5;0.0) | 0.3 (0.0;0.7) |
| Late childhood | *259* |  | **Base level** | -0.2 (-0.5;0.1) | 0.3 (-0.1;0.6) |
|  |  |  |  |  |  |
| The pathway model |  |  |  |  |  |
| Prior to adjustment for lifestyle and adult SEP | *259* |  | **Base level** | -0.1 (-0.4;0.1) | **0.4 (0.1;0.7)** |
| After adjustment for lifestyle and adult SEP | *234* |  | **Base level** | -0.2 (-0.5;0.1) | 0.3 (-0.1;0.6) |
|  |  |  |  |  |  |
| The social mobility model | *259* |  |  |  |  |
| Adult educational level: High |  |  | -0.1 (-0.3;0.1) | 0.1 (-0.1;0.2) | 0.2 (-0.1;0.5) |
| Adult educational level: Average |  |  | 0.3 (0.0;0.6) | 0.4 (0.1;0.7) | 0.5 (0.3;0.8) |
| Adult educational level: Low |  |  | 0.7 (0.3;1.1) | 0.8 (0.5;1.2) | 0.9 (0.5;1.4) |
|  |  |  |  |  |  |
| Separate upward mobility coefficient |  | -0.4 (-0.8;0.1) |  |  |  |
| Separate downward mobility coefficient |  | -0.1 (-0.6;0.5) |  |  |  |
|  |  |  |  |  |  |
| The cumulative model | *248* |  |  |  |  |
| Regression coefficient |  | **0.1 (0.0;0.1)** |  |  |  |
|  |  |  |  |  |  |
| 0-2 |  | **Base level** |  |  |  |
| 3-5 |  | -0.1 (-0.4;0.2) |  |  |  |
| 6-8 |  | **0.5 (0.2;0.8)** |  |  |  |
